# The Virtual Insect Brain protocol: creating and comparing standardized neuroanatomy

**DOI:** 10.1186/1471-2105-7-544

**Published:** 2006-12-29

**Authors:** Arnim Jenett, Johannes E Schindelin, Martin Heisenberg

**Affiliations:** 1 Lehrstuhl für Genetik und Neurobiologie, Biozentrum, Am Hubland, D-97074 Würzburg, Germany

## Abstract

**Background:**

In the fly *Drosophila melanogaster*, new genetic, physiological, molecular and behavioral techniques for the functional analysis of the brain are rapidly accumulating. These diverse investigations on the function of the insect brain use gene expression patterns that can be visualized and provide the means for manipulating groups of neurons as a common ground. To take advantage of these patterns one needs to know their typical anatomy.

**Results:**

This paper describes the Virtual Insect Brain (VIB) protocol, a script suite for the quantitative assessment, comparison, and presentation of neuroanatomical data. It is based on the 3D-reconstruction and visualization software Amira, version 3.x (Mercury Inc.) [1]. Besides its backbone, a standardization procedure which aligns individual 3D images (series of virtual sections obtained by confocal microscopy) to a common coordinate system and computes average intensities for each voxel (volume pixel) the VIB protocol provides an elaborate data management system for data administration. The VIB protocol facilitates direct comparison of gene expression patterns and describes their interindividual variability. It provides volumetry of brain regions and helps to characterize the phenotypes of brain structure mutants. Using the VIB protocol does not require any programming skills since all operations are carried out at an intuitively usable graphical user interface. Although the VIB protocol has been developed for the standardization of *Drosophila *neuroanatomy, the program structure can be used for the standardization of other 3D structures as well.

**Conclusion:**

Standardizing brains and gene expression patterns is a new approach to biological shape and its variability. The VIB protocol provides a first set of tools supporting this endeavor in *Drosophila*. The script suite is freely available at [2]

## Background

Attempts to assign behavioral and mental functions to regions of the brain are as old as brain science. Today, in insects this mapping attempt reaches the resolution of neurons and neuronal circuits. In particular, the 2-component Gal4 technique in *Drosophila *[3] facilitates this approach providing the means for manipulating small neuronal assemblies *in vivo*. These can be visualized and their properties be changed, even in a temporally controlled manner [4]. Driver lines exhibiting a particular expression pattern of the yeast transcription factor GAL4 are combined with a GAL4-controlled transgene of choice, and those flies, then, are subjected to behavioral, physiological or anatomical analysis. As the neuronal assemblies of gene expression patterns often are complex, their usefulness for functional studies depends upon their rigorous anatomical analysis.

The constancy and variability of biological shapes can be quantitatively assessed by standardization. This approach has been introduced to the study of the *Drosophila *brain by Rein et al. [5,6] who provided a prototype of the VIB protocol with limited functionality. Due to the generation and increasing use of large collections of GAL4 driver lines in the last years, improved tools for their anatomical investigation are at high demand. We have now developed the Virtual Insect Brain (VIB) protocol serving a variety of applications in invertebrate neuroanatomy. For instance, and most significant in *Drosophila*, the biological variability of gene expression patterns can be measured and evaluated by standardization. Standardized expression patterns can be directly superimposed. Their common and different parts can be identified in the overlay, even if they are complex.

## Implementation

The VIB protocol was developed for standardizing and comparing the shapes of *Drosophila *brains and the gene expression patterns within these brains. The protocol uses data sets of 3D reconstructions of brains obtained from immunostained wholemount preparations by confocal microscopy. The protocol is limited only by the size of the data sets. The protocol can cope with this limitation by applying a user-defined step of resampling to the data sets. It can hence be used for the standardization of any other biological 3D shapes. Considering biologists without extra computing experience to be the main users, we introduced all functions of the VIB protocol to the Amira graphical user interface (GUI) and constructed a dynamic configuration script which enables the user to set up the parameters of the process at the start. No further configuration is necessary during the process of standardization.

### Definitions

#### Template

The Template data set consists of a 3D image and the corresponding LabelField (see next paragraph). It provides the common coordinate system to which the selected brains get registered. For *Drosophila melanogaster *(wildtype Canton S) a Template for each gender is provided The Template needs to display the most normal brain morphology, which is selected in a process described below (see Templates; page 12).

#### LabelField

A LabelField is a regular cubic grid with the same dimensions as the underlying 3D image. For each voxel it contains a label indicating the region the voxel belongs to [7]. LabelFields are created automatically by the VIBlabelWizard (see Labeling, page 9) and saved with the name of the corresponding 3D images in the labels folder (VIB/labels). With respect to their contents, the labeled neuropil regions are called Materials. Their volumes, surfaces and centers of gravity are used to compute rigid and non-rigid transformations of the Samples (see Registration, page 10).

#### Sample

The Samples are the 3D images of a file group assembled for standardization (see File group:, page 6). Using the VIBregistrationTransformation (RT) 3D images are sufficient for the Samples. All other transformations need LabelFields which are produced while carrying out the VIB protocol (see Labeling, page 9).

### Data import and export

The VIB protocol creates its own folder structure during installation inside its root folder ([Installation directory]/VIB). The raw 3D image data to be standardized have to be introduced to the protocol by placing them to the images folder (VIB/images). Alternatively to copying those to VIB/images on UNIX systems symbolic links can be created in VIB/images pointing to the actual storing position of the images.

#### Supported image file formats

Using the import algorithms of Amira the VIB protocol principally can work on all image file formats supported by Amira (see table Fig. 7).

The VIB protocol was developed and extensively tested on stacked tagged image files (tif) created by a Leica-SP1 confocal microscope with a resolution of 8 bits. This file format was chosen because it is a common format most confocal microscopes are able to produce. Nevertheless proprietary image file formats like .pic (BioRad) or .lei (Leica) were tested successfully.

#### Results

Results of the standardization are saved in the output folders (VIB/output), sorted by type

- non-rigidly transformed images → VIB/output/warped

- average images → VIB/output/average

- Main probability maps → VIB/output/Mainprob

- Material's probability maps → VIB/output/prop

#### Statistics

Besides the image data and their derivates statistical data are saved in the statistics folder (VIB/statistics). Together with the internal statistics files in proprietary Amira file format (AmiraMesh Format (.statistics)) all statistics are exported as comma separated values (.csv) for further analysis in any spreadsheet software (VIB/statistics/csv). The VIB protocol creates a table of statistical data for each image file respectively its LabelField (see QV VIBtissueStatistics) and two additional tables a containing basic statistical analysis for each file group's LabelFields.

### Configuration

**Figure 1:**
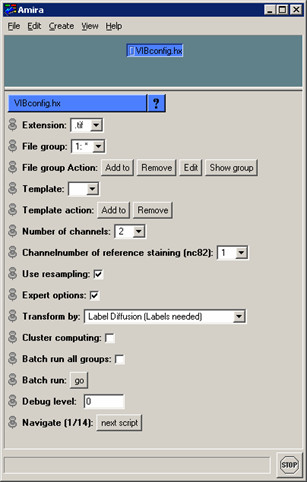
**VIBconfig.hx**. The configuration script of the VIB protocol in maximum extent. Most of the functions are dispensable for normal standardization. Expert options are implemented for extended automation of the protocol.

The VIB protocol is solely configured in the script VIBconfig.hx (Fig. 1) which is called at the start of the protocol. It is recommended to fill in the form from top to bottom because the VIBconfig.hx is a dynamic script which changes its options according to earlier input. Before the configuration can be executed the confocal 3D images to be standardized have to be inserted in the images folder described above (VIB/images). In the following the VIBconfig.hx is described line by line.

#### Extension

This multi-menu displays all file extensions located in the images folder (VIB/images). The VIB protocol is restricted to work on one image file format per file group (see next paragraph) only during standardization. This has to be selected here.

#### File group

As a statistical approach to neuroanatomy the VIB protocol works on groups of 3D image files representing the same type of brain. All of these files are supposed to be located in the images folder and can be grouped using the 'File group Action' buttons. Multiple file groups can be set up to make batch runs (see Batch run:, page 8) more effective.

#### Template

If there is no LabelField for the Template 3D image in the first run the Template can be defined later on in the VIBlabelWizard. Templates can be added using the 'Template action' buttons.

#### Number of channels

As the VIB protocol works on multi-channel tagged image files (.tif), the number of channels included in a raw 3D image file has to be defined as well. If the number of channels is set to >1, a new line appears in which one of the channels has to be defined as the 'reference channel'.

#### Use resampling

Regular 32-bit computers today are not capable of processing the VIB protocol on big data sets (>> 512 × 512 pixels per slice). Therefore data sets bigger than 512 × 512 pixels should be resampled. Test runs on a 64-bit system (Onyx2 InfiniteReality) proved that the step of resampling is dispensable on high-end computers.

Because the step of resampling is done after the segmentation the interactive processes (VIBscissors, VIBlabelWizard) can be accomplished at high resolution. These steps are all that is needed for non-experts to configure the VIB protocol.

#### Expert options

Checking 'Expert options:' unfolds advanced configurations. When activated these allow for enhanced configuration of the VIB protocol. In this section the transformation method can be changed as well as cluster computing and batch run can be enabled.

#### Transform by

In addition to the pre-set Transformation (i.e. VIBdiffusionTransformation, see Registration, pages 10ff) three other transformations are implemented in the VIB protocol and can be selected via this multi-menu.

#### Cluster computing

To speed up the work on very large file groups, one can process the files on several computers running the VIB protocol in parallel. Internal blocking of the current file while it is processed prevents redundant computation in the cluster if 'cluster computing' is enabled. For blocking the VIB protocol writes lock files into the corresponding folders containing only the name of the blocking computer. These text files are named with the base name of the file to be blocked and a file name extension created from the name of the blocking module with the suffix "LCK". (e.g. imageA.splitLCK is the lock file of the file 'imageA', created by the module VIBsplitChannels). Checking for these files before upload enables different modules to work with the same data, but redundant processing is avoided. Additionally, the suffix "LCK" simplifies the retrieval of orphaned lock files after a potential software crash.

#### Batch run all groups

In the ground state of the protocol the batch run is confined to the current file group. The batch run can be extended to all file groups following the selected file group by enabling this option.

#### Batch run

The VIB protocol can be run automatically if all LabelFields already exist or LabelFields are not needed (Amira's Registration). Batch run is started with this button

#### Debug level

This is a switch between three levels (0–2) of verbosity of the VIB protocol seen in the Amira console. This output also is appended to the log.txt file needed for debugging. At levels >0, the 'Jump to Script' menu appears which enables direct selection of any script included in the configured procedure.

#### Navigate

The 'Navigate' buttons lead through the process of standardization by loading the appropriate scripts and files. The progress of the protocol is displayed by the number of the current script next to the total number of scripts needed.

### Processing

**Figure 2:**
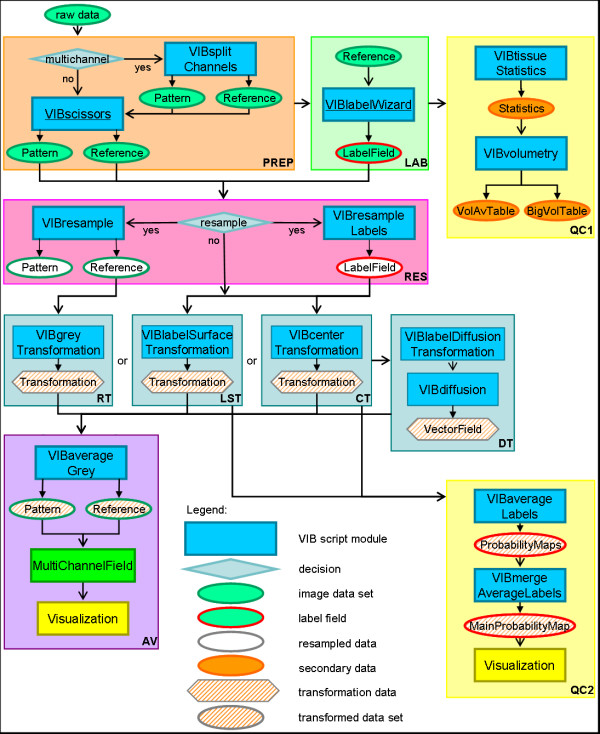
**Flowchart of the VIB protocol**. The process starts with the import of raw data (upper left). Sections: PREP (light brown): In the steps of preprocessing the imported 3D images are prepared for standardization. These steps include optional resampling and electronic removal of unwanted parts of the 3d images. LAB (light green): During the labeling LabelFields are constructed on the basis of the reference channel; QC1 (light yellow): The first quality control evaluates the LabelFields by basic statistical comparisons of the Material's volumes; RT, LST, CT, DT (light blue): The transformation processes align the Samples to the Template rigidly (RT, LST, CT) or non-rigidly (DT); QC2 (light yellow): The second quality control for the evaluation of the alignment creates MainProbabilityMaps for each file group. These can be controlled visually and statistically; AV (purple): The step of averaging fuses each channel of the 3D images of a file group to an average intensity image. Icons: Ovals: data; squares: processes; diamonds: decisions; hexagon: transformations. Color code: green: 3D images; red: LabelFields; blue: processes/scripts; orange: spreadsheets; yellow: visualization; white: resampled data; hatched: transformed data. Arrows: open: data input to processes; closed: writing data to files.

To register and standardize confocal 3D images of neuropil structures and corresponding GAL4 expression patterns, they are processed by the VIB protocol in a robust sequence of steps (Fig. 2). For the *Drosophila *brain normally a ubiquitously expressed neuropil marker such as bruchpilot (MAK nc82) is used as reference. 3D image data of more selective gene expression patterns may be standardized in the additional channels.

#### Preprocessing

To cope with different file formats and high resolution files the raw data (Fig. 2, 'raw data') have to be preprocessed before being fed into the process of alignment and standardization. For multi channel image data (e.g. Leica tiff) preprocessing (Fig. 2, PREP) starts with the splitting of the data sets into single channel files by VIBsplitChannels (Fig. 2). According to the transformation method, selected tissues attached to or accompanying the brain can be manually removed in the 3D images (Fig. 2, PREP, VIBscissors). This 'cleaning' procedure reduces potential artifacts in subsequent alignment steps.

#### Labeling

Standardization procedures based on the volumes and shapes of selected neuropil structures require manual segmentation. This is done in the VIBlabelWizard (Fig. 2, LAB) resulting in LabelFields. These represent the neuropil structures of interest. For segmentation the 3D images of the reference channel are used in original resolution to attain highest accuracy of the LabelFields. If the original x-/y-dimensions of the raw data are bigger than 512 × 512 pixels the LabelFields are automatically resampled after segmentation is completed (VIBresampleLabels). The resampled LabelFields are stored in a new directory to be used in the following processes (Fig. 2, RES).

#### Quality control

Two routines for quality control (Fig. 2, QC1/2) are implemented in the VIB protocol's statistical methods and visualization. The first one (Fig. 2, QC1) enables the user to evaluate the LabelFields by providing a set of spreadsheets containing statistical data on the labeled regions. For the user's convenience these data are arranged in two spreadsheets to simplify file to file comparisons (VIBvolumetry). These files containing 'comma separated values' (csv) are located in the folder VIB/statistics/csv/ and can be analyzed with any spreadsheet application.

**Figure 3:**
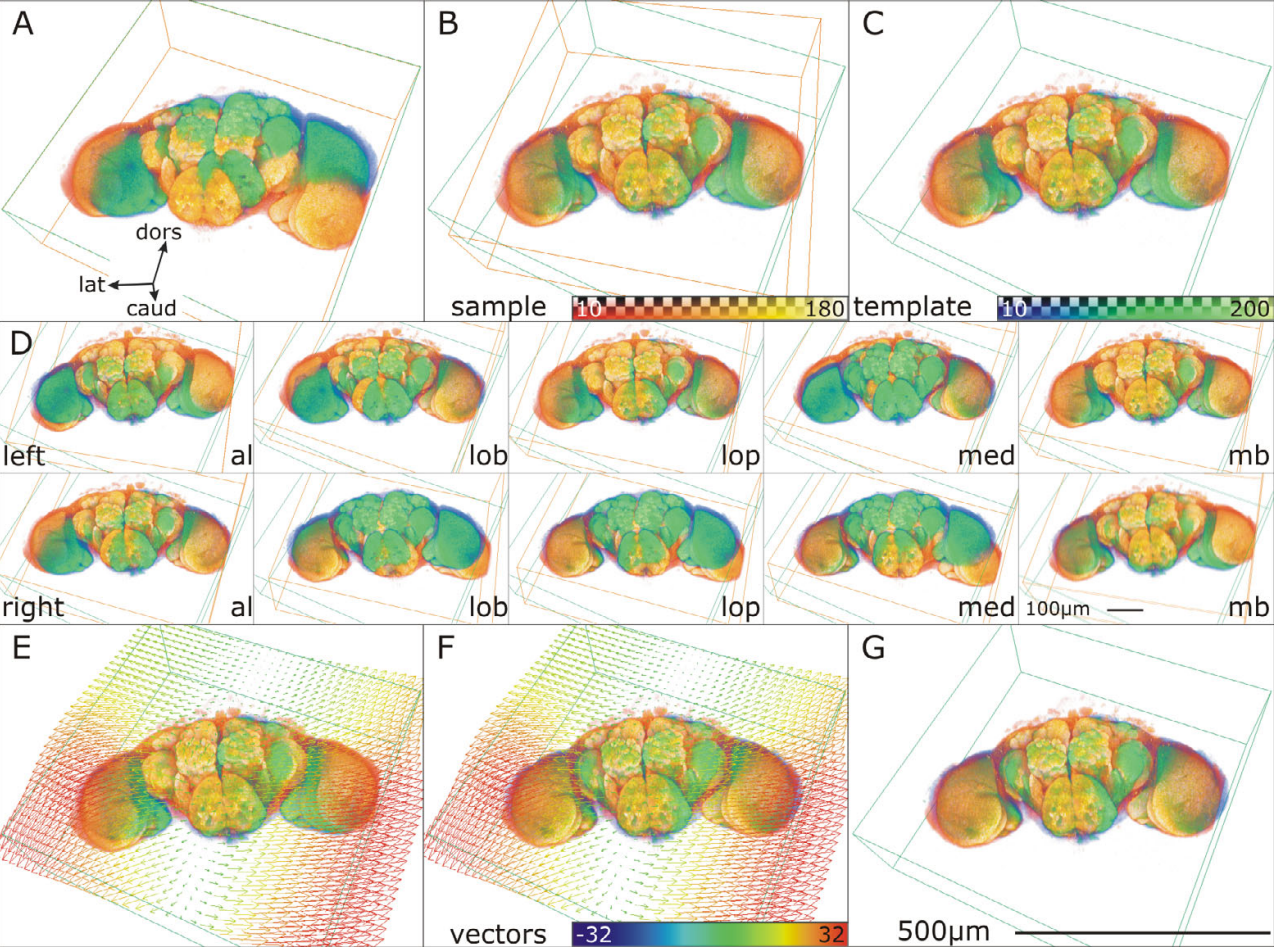
**Registration of two *Drosophila *brains**. Template: blue/green, Sample: orange. A: Template superimposed with untransformed Sample. B: Global rigid transformation applied to the Sample. C: Result of rigid transformation: Sample transferred to Template coordinate system after rigid transformation (VIBcenterTransformation.hx). D: Local rigid transformation for 10 labeled neuropil regions (VIBlabelSurfaceTransformation). For each Material (al: antennal lobe, lob: lobula, lop: lobula plate, med: medulla, mb: mushroom body) the overlap with the corresponding Template Material is maximized without taking the other Materials into account. Thereby as many contradictory rigid transformations are generated as Materials are defined. E: VectorField of non-rigid transformation superimposed. F: non-rigid transformation applied to Sample (VIBdiffusion.hx). G: Result of non-rigid transformation: Template superimposed with transformed Sample. Surrounding boxes outline the data volumes and coordinate systems.

The second step of quality control (Fig. 2, QC2) is conducted after transformation of the LabelFields. It provides a visual test to control for the quality of the transformation applied by the label based transformations (see Registration, page 10). The transformation parameters are applied to the LabelFields of the selected file group individually using the Amira module 'Average Brain' (VIBaverageLabels). The resulting Probability Maps (Fig. 3) indicate the spatial distribution of the probability of a Material's occurrence. These are saved and fused by VIBmergeAverageLabels to generate a MainProbabilityMap. These files can be used to evaluate the results of standardization by visualizing them in false colors (Fig. 3, 4b–d). The thinner the seam is between the probability of 100% (red) and 0% (blue), the smaller is the variance in the standardization. This evaluation can be quantified using the P75-hull-to-core-ratio (see Hull-to-core-ratio, page 13).

**Figure 4:**
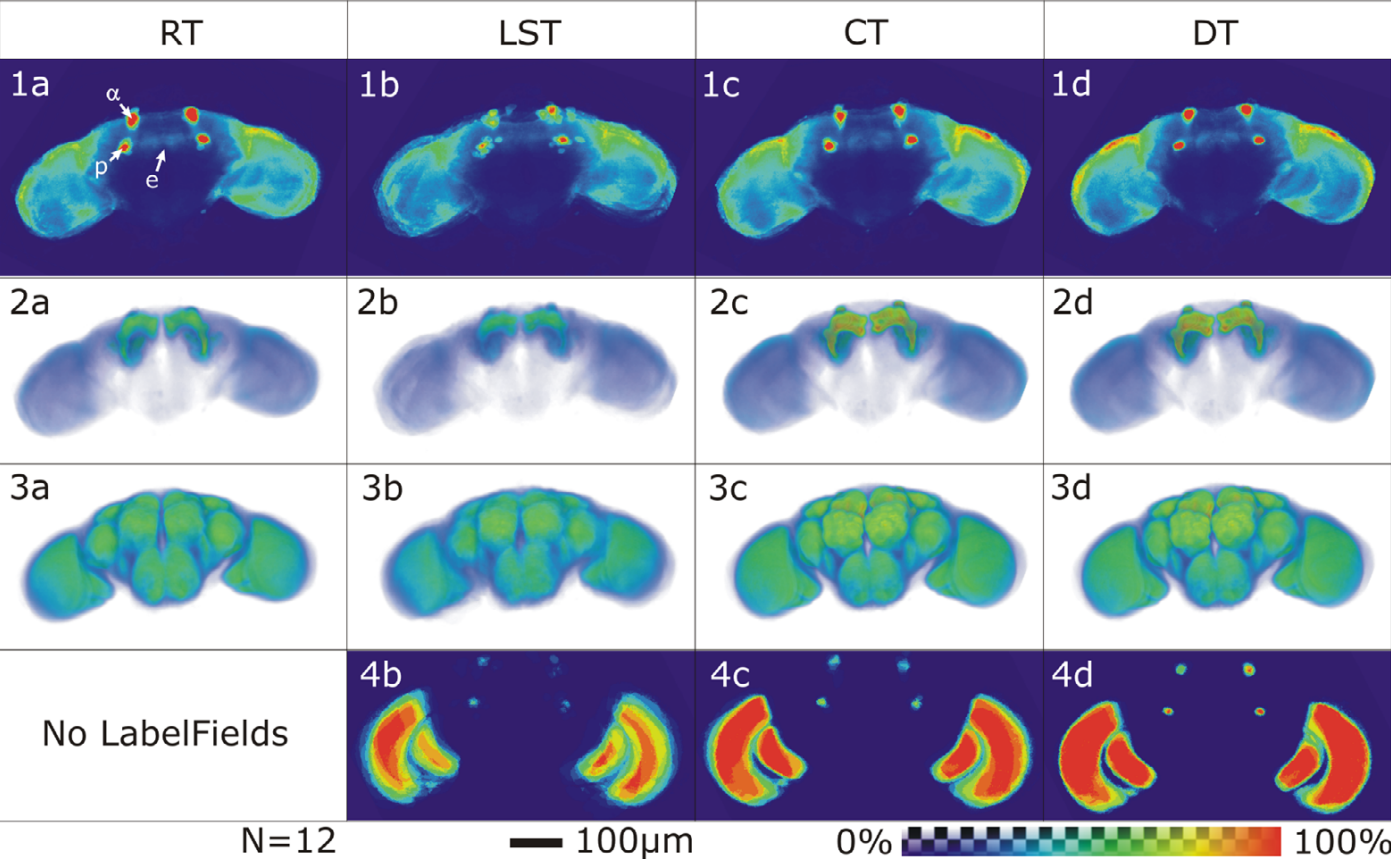
**Comparison of Standard Brains created with four different standardization algorithms**. Average intensity maps of the pattern channel (line 1+2) display the typical expression pattern of the Gal4 driver line, while average intensity maps of the reference channel (line 3) display the typical morphology of the file group, visualized using the antibody nc82. Brightness is coded in false colors. The difference in fuzziness and the volume of uniformly bright areas can be taken as a measure for alignment quality. MainProbabilityMaps reflect the distribution of the Materials of a file group after transformation (in %). False color coded areas with maximum probability of occurrence of a Material (Material overlaps in all Samples, core) are red, areas outside Materials are blue (see false color scheme). Using the same set of data, one can take the ratio between the core and the area of low probability of occurrence of a Material (seam) as a measure of transformation quality. No MainProbabilityMap is generated for the AmiraRegistrationTransformation because this method does not use LabelFields for the alignment of the Samples but the distribution of the grey values in the 3D images. 1^st ^line: (1a-d): Medial frontal section through a standardized Gal4 expression pattern. The section lies posterior to the horizontal lobes, crosses the tip of the α/α'-lobes (α) the peduncle (p) and the ellipsoid body (e), which is not stained in 201y. 2^nd ^line (2a-d): Direct volume rendering of standardized Gal4 expression patterns. 3^rd ^line (3a-d): Direct Volume Rendering of standardized nc82-reference patterns. 4^th ^line (4b-d): MainProbabilityMap at the same level as 1b-d. The uppermost dots represent the tip of the α/α'-lobes, the dots beneath are cross sections of the mushroom body peduncles. In the optic lobes the section runs through the medulla, the lobula and the very tip of the right lobula plate. Figs. 1-3: Averaged intensities color coded. Figs. 4b-d: Material's probabilities of occurrence color coded. n = 12. Flies: female Gal4-201y × UAS:mcd8GFP.

#### Resampling

High resolution data larger than 512 × 512 pixels in x-/y-dimension have to be resampled before being fed into the standardization process (Fig. 2, RES, VIBresample). On future computer systems this step of preprocessing will become dispensable and therefore it can be switched off during configuration (VIBconfig.hx).

#### Registration

As mentioned above, four transformation algorithms can be chosen in the configuration step (VIBconfig.hx; light blue boxes in Fig. 2). Three of them (RT, LST, CT) are rigid (translation and rotation only) implying that during transformation the relative positions of points in the Sample are not changed. During the DT sub areas of the Samples are aligned individually resulting in a non-rigid transformation.

**VIBregistrationTransformation (RT) **is a global rigid transformation based on principle component analysis (PCA) of the grey values of the 3D images. Using the Amira module 'Registration' rigid transformation of the file groups data sets onto the Template data set is computed, using an iterative optimization algorithm [7]. This process correlates the grey values of the 3D images allowing isotropic scaling of the Sample. Thereby the scaling is applied after rotation and translation of the Sample. Thus scaling artifacts can be avoided to a large extent.

The **VIBlabelSurfaceTransformation (LST) **is a global rigid transformation based on the functionality of the Amira module 'AlignSurfaces' [7]. The LST generates a triangular approximation of the interfaces between the Exterior and the Interior of all the Materials defined in the LabelFields of the Sample and the Template using the Amira module 'SurfaceGen'. Surfaces are aligned in their entirety to each other by iteratively minimizing the root mean square distance between corresponding surfaces [7]. This process is called the iterative closest point algorithm (ICP) [8].

The **VIBcenterTransformation (CT) **computes a global rigid transformation of the Samples on the basis of the centers of gravity of the Materials. These are stored in the corresponding statistics files by the script VIBtissueStatistics. The script creates landmark sets from the coordinates of the neuropils' centers of gravity. Landmark sets represent specific points or markers in 3D space. On the basis of these data sets a rigid transformation is computed by minimizing the sum of the squared distances between corresponding points. I.e., the script moves the points of the Sample as close as possible onto the points of the Template without changing their relative position to each other. The result is returned as a 4 × 4 transformation matrix and written to the corresponding statistics file.

For computing a non-rigid transformation the **VIBdiffusionTransformation(DT) **is the most costly but most precise transformation in the VIB protocol. It consists of three steps. Beginning with the algorithms of the CT a global rigid transformation of the Sample onto the Template is applied, followed by a local rigid transformation (VIBlabelDiffusionTransformation). In this step for each of the LabelFields the 3D overlap of corresponding Materials is maximized without taking into account the degree of overlap of the other Materials using parts of the functionality of the LST. By this as many contradictory transformations are computed as Materials are defined in the LabelFields. In the last step (VIBdiffusion) the contradictory transformations are applied to their corresponding Materials and non-labeled areas in between are transformed following a modified heat transfer equation [6]. This step results in a VectorField which is directly applied to the 3D images. The non-rigidly transformed 3D image files are saved in the VIB/output/warped folder from where they are recalled by the process of standardization.

#### Standardization

In contrast to the non-rigid transformation of the DT the rigid transformation parameters are stored in the statistics files. From there they are applied to the 3D images (VIBaverageGrey). On the basis of the transformed 3D images a three dimensional average image is computed. This is the standard brain (average; AV). If multi-channel images are used the standard brain is accompanied by standardized gene expression patterns. These files are stored in the VIB/output/average/folder and can be uploaded to Amira to be visualized simultaneously using MultiChannelField.

## Results and discussion

The VIB protocol has been developed on the basis of the script suite originally used to compute the *Drosophila *StandardBrain [6]. The present version minimizes the users' interaction with the underlying program structure. This saves time. The hazard of losing data is reduced by systematically saving all intermediate results. Every script of the VIB protocol starts with a check of data integrity. This allows for direct resumption of the standardization at the step of a potential computer crash.

### Templates

The process of standardization is dependent on a Template representing a reference (e. g. wild-type) brain. For *Drosophila melanogaster *we provide a female and a male Template data set of CantonS wild type. These were selected out of 40 female and 39 male brains. To avoid bias in the selection of the primary Template we refrain from the iterative approach of Template selection described earlier [6] &[9]. The following two absolute measures were developed for the selection of the Templates, the overall mean distance of centers of gravity (mean-cog-distance) and the P75-hull-to-core-ratio (P75-ratio).

#### Overall mean distance of centers of gravity

The Overall mean distance of centers of gravity (mean-cog-distance) describes the (overall) mean distance of all Materials' centers of gravity of the Template to the corresponding centers of gravity in the transformed data sets. The smaller this absolute measure, the more 'normal' is the current Template for the selected group of brains. For each data set of the selected file group the mean-cog-distance is calculated by using it as the Template for the file group. Using global rigid transformation, deformations or aberrations from normal morphology in the current Template become obvious and result in a large mean-cog-distance. In a pre-screen we computed the mean-cog-distance on groups of 40 female and 39 male brains.

#### Hull-to-core-ratio

For control reasons ten data sets with the smallest mean-cog-distance were introduced in this volumetric measure based on non-rigid transformation. The P75-ratio is applied after the LDT was processed on the basis of the MainProbabilityMap (see above). Thereby the areas of low probability (hull; P < 75%) are separated from those with high probability (core; P > = 75%). The mean of the ratios core/hull of a file group describes the suitability of the current Template.

Both methods of Template selection lead to congruent results.

### *Drosophila *template data set

For the standardization of the *Drosophila melanogaster *brain (fused supra- and subesophageal ganglion) we selected one female and one male 'data set' as Template. They are downloadable as compressed files (zip) [2]. Extracting these to the VIB root folder will bring them to the right starting position in the VIB folder tree. The Template provides the common coordinate system on which all standardizations in our lab are based. For comparison we constructed two standard brains derived from 40 female and 39 male specimens stained with nc82.

### Comparisons of different expression patterns

**Figure 5:**
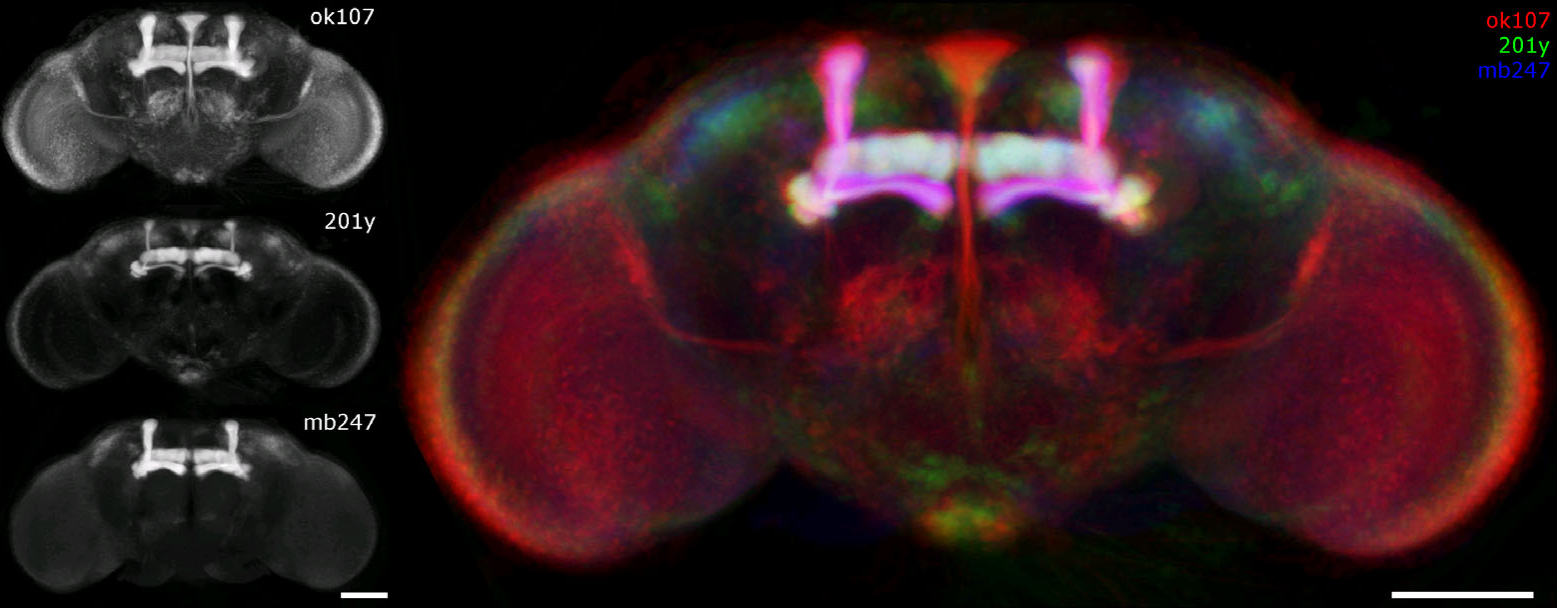
**Comparison of standardized expression patterns**. Projection view on standardized expression patterns (ok107/201y/mb247). Gal4 lines were crossed to UAS-EGFP2 for visualization, scanned with a Leica-SP1 confocal microscope (8-bit tifs) and standardized using VIBdiffusionTransformation(DT). On the left the standardized expression patterns are visualized in grey values (N_ok107 _= 17, N_201y/mb247 _= 15). On the right the same images are superimposed and color coded (ok107 red, 201y green, mb247 blue). In structures common to all three expression patterns colors add up to white (e.g. γ-lobes of the mushroom body Gal4-positiv in all lines displayed (white), α'/(β') and medial bundle Gal4-positive in ok107 only (red), besides strong Gal4 expression in the pars intercerebralis (PI) in ok107 some cell bodies are Gal4-positive as well in 201y (green)), Scale bar 100 μm

An example of the comparison of three different Gal4 lines with strong expression in the mushroom bodies (201y, ok107, mb247) is given in Fig. 5. Visual analysis of differences and common aspects of the standardized expression patterns can easily be accomplished using the Amira visualization tools [9] in combination with the Amira MultiChannelField (MCF). Alternatively the surfaces of the standardized expression patterns can be constructed by thresholding the data sets (Amira Isosurface [7]). This module divides the voxels of the current data set into two groups according to their brightness and triangulates an Isosurface at the groups' interface. Using different color maps the standardized expression patterns can be conveniently filtered from background noise.

### Analysis of interindividual variability or individual expression patterns

For the analysis of individual Samples the transformed 3D images are saved in the folder VIB/output/warped. Visualizing these using the Amira MCF enables direct comparison of unique data sets. After standardization stained structures of backfill studies or single cell stainings (MARCM; [10]) can be easily superimposed and analyzed. In the same manner individual expression patterns can be analyzed and compared to any standardized structure.

## Conclusion

The VIB protocol is a new tool for the quantitative comparison of 3D shapes. It can be used without any programming experience, providing an interactive graphical user interface. Four different registration methods are implemented allowing for different degrees of standardization. Using the VIBregistrationTransformation (RT), standardization can be quickly achieved with no need for the time-consuming step of manual labeling which is needed for all other approaches. While RT only results in a crude approximation of the samples to the Template, it can provide a quick comparison of the overall neuroanatomy and of expression patterns.

As RT, the VIBlabelSurfaceTransformation (LST) and the VIBcenterTransformation (CT) are global rigid transformations. Their precision of standardization is highly dependent on the quality of dissection and uniformity of the specimens. They can be used for comparing broad arborization patterns.

As a non-rigid transformation the VIBdiffusionTransformation results in the most precise standardization. Single cell analysis (e.g. MARKM) should only be performed using this standardization technique.

All standardization results can be evaluated by statistical control mechanisms like spreadsheets of extracted volumes and main probability maps of the labeled regions.

## Methods

### Immunochemistry

Flies were raised under standard conditions and etherized before preparation. Brains were dissected under cooled phosphate buffered saline (PBS) and fixed in pre-cooled 2% paraformaldehyde (PFA) overnight at 4°C. To permeabilize the tissue all washing or incubation was conducted in PAT (PBS plus 1 g/100 ml bovine albumin (Sigma, A6793), 0,5% Triton X 100(Sigma, X-100). After removal of the PFA (3 × 20 mins PAT) specimens were blocked with 3% normal goat serum/PAT for 1 h at room temperature. Excess blocking solution was removed and specimens were incubated with 1:1000 dilution of highly cross absorbed anti-green fluorescent protein antibody (A-6455, MoBiTec, Goettingen, Germany) in PAT overnight at 4°C. Specimen were thoroughly washed in PAT and then incubated with 1:10 dilution of nc82 [11] monoclonal antibody overnight at 4°C.

Secondary antibodies were goat anti-mouse F(ab')2 coupled to indocarbocyanine fluorophore Cy3 (Jackson Immuno Research, West Grove, PA) diluted 1:250 and highly cross-adsorbed Alexa Fluor^® ^488 goat anti-rabbit IgG (H+L) (A-11034, MoBiTec, Goettingen, Germany) diluted 1:100 in PAT. Incubation was overnight at 4°C. To reduce background staining the specimens were washed in PAT for the next three days with gentle agitation at 4°C. The specimens were mounted in Vectashield diluted 3:1 with PBS.

### Confocal microscopy

Whole-mount brains were scanned with a Leica TCS-NT confocal microscope, equipped with a Ar/Kr laser and a Leica Pl Apo 20X NA 0.7 IMM lens. Frontal series of entire brains were taken each μm in z-direction with 1024 × 1024 pixel resolution using an average zoom of 0,8 to make the specimen fit to the optic field. The resulting optical resolution is 0,6 × 0,6 × 1 μm^3^. For scanning wholemount brains of *Drosphila melanogaster *with these settings, one obtains image stacks of about 200 ± 20 slices. Fluorochrome excitation was set to 488 and 568 nm, emitted fluorescence signals were detected in the range of 493–559 and 607–701 nm.

### Quality test for reliability

**Figure 6:**
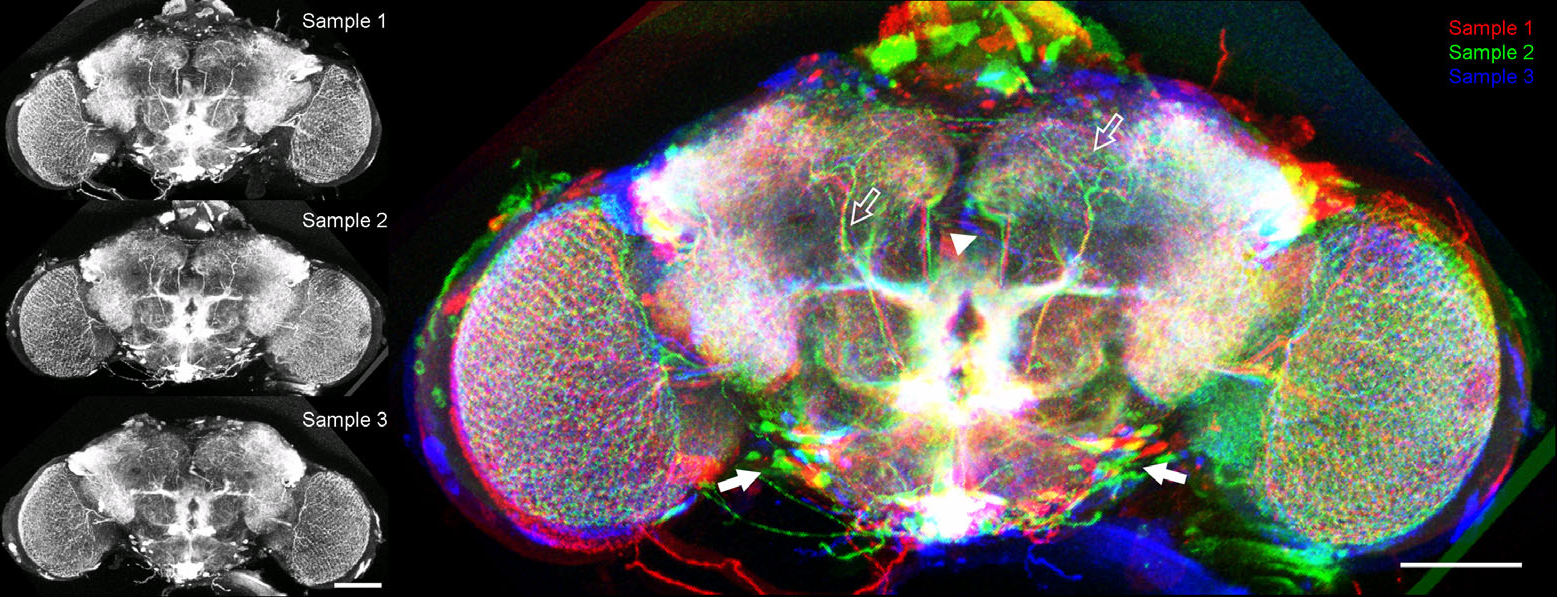
**Comparison of interindividual variability in Gal4 expression patterns**. Projection view on three individual expression patterns standardized to the same Template. Gal4-NP7088 was crossed to UAS:mcd8GFP. Invariable parts in the distribution of the expression patterns in the central brain (open arrows) easily can be discerned from variable parts as the cell body layer (closed arrows). The resolution and the precision of the transformation are high enough for single cell analysis. Anomalies of single neurons like in Sample 3 (blue) become obvious by this technique (arrowhead). Scale bar 100 μm

During the development of the VIB protocol all methods were tested extensively for reliability using several test settings. The current version of the VIB protocol yields perfect results in repeatability and accuracy of transformation (see Fig. 6) in all tests applied.

**Figure 7:**
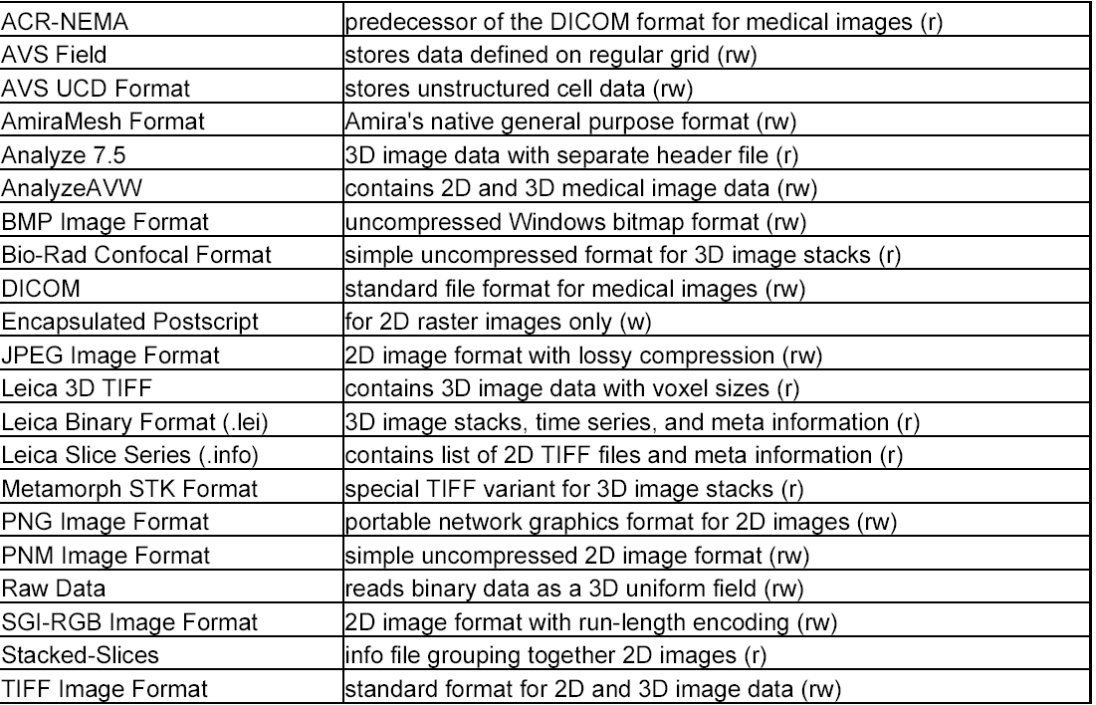
**Alphabetic index of image file formats supported by Amira (extracted from Amira user's guide)**. Listed image formats are supported by Amira and therefore can be used with the VIB protocol. The letters in brackets stand for the processing abilities (read, write) of Amira.

### Availability and requirements

Project name: Virtual Insect Brain Protocol

Project home page: 

Operating system(s): IRIX, Linux, Windows

Programming language: Tcl, C++

Other requirements: Amira 3.x

License: free

Any restrictions to use by non-academics: non

## Abbreviations

PREP Preprocessing

LAB Labeling/Segmentation

QC Quality control

RES Resampling

RT AmiraRegistrationTransformation

LST VIBlabelSurfaceTransformation

CT VIBcenterTransformation

DT VIBdiffusionTransformation

AV Standardization

PCA principle component analysis

ICP interactive closest point

201y GAL4-201Y

ok107 GAL4-OK107

mb247 P{GAL4}247

PBS phosphate buffered saline

PAT PBS plus bovine albumin and TritonX-100

PFA Paraformaldehyde

al antennal lobe

lob lobula

lop lobula plate

med medulla

mb mushroom body

## Authors' contributions

MH managed the overall project. JES and AJ conceived the software architecture. JES wrote most of the software code. AJ wrote the documentation and the manuscript and participated in testing and coding the graphical user interfaces. All authors read and approved the manuscript.
